# Investigating the Frequency of Placenta Previa and the Associated Risk Factors During Pregnancy

**DOI:** 10.7759/cureus.86053

**Published:** 2025-06-15

**Authors:** Zoha Asim, Kashmal Khattak, Eeman Ali, Nabisha Zia, Maahin Ali, Rukhsana Gul, Farid Ullah, Tahira Bibi, Hizb Ullah, Arif Ullah Khan

**Affiliations:** 1 Obstetrics and Gynaecology, Tehsil Headquarter Hospital (THQ) Bazar Zakha Khel, Khyber, PAK; 2 Obstetrics and Gynaecology, Medical Teaching Institute - Hayatabad Medical Complex, Peshawar, PAK; 3 Surgery, Lady Reading Hospital, Peshawar, PAK; 4 Surgery, Khyber Medical College, Peshawar, PAK; 5 Community Medicine, Khyber Medical College, Peshawar, PAK; 6 Obstetrics and Gynaecology, Ayub Teaching Hospital, Abbottabad, PAK; 7 Medicine, Khyber Teaching Hospital, Peshawar, PAK; 8 Obstetrics and Gynaecology, Khalifa Gul Nawaz Teaching Hospital, Bannu, PAK; 9 Medicine, Bahria University Health Sciences Campus Karachi, Karachi, PAK; 10 Surgery, Ayub Teaching Hospital, Abbottabad, PAK

**Keywords:** cesarean section, multigravidity, multiparity, placenta previa, vaginal bleeding

## Abstract

Introduction: Placenta previa (PP) is the abnormal implantation of the placenta over the lower uterine segment, either partially or completely covering the internal os. It is a major cause of maternal and fetal morbidity and mortality. This study aimed to determine its frequency and evaluate the risk factors for PP among pregnant women.

Methodology: This cross-sectional study included 142 pregnant women, selected through non-probability consecutive sampling. Data on demographics, obstetric history, and risk factors were collected using a structured proforma. Analysis was performed with IBM SPSS Statistics, version 22.0 (IBM Corp., Armonk, NY), describing categorical variables with frequencies and percentages. Associations were evaluated using the chi-square test, with a p-value of ≤0.05 considered significant. Quantitative variables such as age, parity, and gravidity were described using mean, median, mode, and standard deviation.

Results: The mean age of participants was 26.33 ± 5.37 years. The frequency of placenta previa was 6.3% (9 out of 142). Most cases (78%) occurred in women under 35 years of age. Placenta previa was significantly associated with multigravidity (p = 0.01), multiparity (p = 0.026), and a past history of PP (p = 0.006). Among women with a prior history of placenta previa, 66.6% experienced recurrence.

Conclusion: This study found a high frequency of placenta previa, with significant risk factors including previous PP, multigravidity, and multiparity. Additional associated factors included previous cesarean sections and maternal age below 35 years. These findings underscore the importance of vigilant antenatal screening for high-risk pregnancies.

## Introduction

The human placenta is a hemochorial and deciduate connection between the mother and fetus that ensures the transport of nutrients and oxygen while removing waste from the fetus through the umbilical cord [1]. Placenta previa (PP) is an abnormal positioning of the placenta, where it is implanted in the lower segment of the uterus, either completely or partially covering the internal os of the cervix [1]. Based on clinical assessment through cervical palpation or imaging, placenta previa is categorized into three types: complete previa, where the placenta entirely covers the internal cervical os; partial previa, where the placenta partially covers the cervical opening; and marginal previa, where the placental edge reaches or is near the internal os but does not cover it. In the UK, it is described as major or minor and is graded from I to IV [2].

Globally, the prevalence of PP is approximately 5 per 1000 pregnancies, rising to 12 per 1000 pregnancies among Asian women and dropping to 3 per 1000 pregnancies in Europe [3]. Placenta previa is a significant cause of maternal and perinatal mortality. Perinatal mortality due to PP is reported as 6.6 per 1000 live births, while maternal mortality remains low in regions such as Jamnagar, Gujarat, India [4]. In Pakistan, the prevalence rate of placenta previa was found to be 6 per 1000 in a study conducted in Combined Military Hospital (CMH) Kharian, Pakistan, from February 2015 to March 2018 [5].

The exact cause of placenta previa is still unknown, but theories suggest that dropping of the fertilized ovum in the lower segment or a defective decidual layer in the upper segment of the uterus could lead to abnormal implantation. Patients typically present with painless, recurrent vaginal bleeding as the only symptom. Vaginal bleeding during the second trimester, confirmed by ultrasound or MRI, is indicative of placenta previa. This condition can lead to various complications, including antepartum, intrapartum, or postpartum hemorrhage, malpresentation of the fetus, and maternal death due to massive hemorrhage. Fetal complications include asphyxia, low birth weight, birth injuries, or intrauterine death [1]. Other neonatal complications associated with placenta previa include neonatal death (280 per 1000 live births), prematurity at birth (42.85%), respiratory distress syndrome (28.4%), and aspiration (14.2%) [6]. Placenta previa is a significant cause of obstetric hemorrhage, and women with complete placenta previa are at an increased risk of requiring blood transfusions during cesarean sections. Independent risk factors for blood transfusion in such cases include previous cesarean sections, complete placenta previa, and preoperative bleeding [7].

Identified risk factors for placenta previa include advanced maternal age, gynecological diseases, alcohol consumption during pregnancy, multiparity, multigravida, previous cesarean sections, scars, or malpresentation of the fetus [8]. Additional risk factors include a history of abortion, surgical evacuation of pregnancy, or postpartum hemorrhage [9]. According to a meta-analysis and systematic review, singleton pregnancies achieved through assisted reproductive techniques (ARTs) have a higher risk of placental anomalies, such as placenta previa and placental abruption, compared to non-ART singleton pregnancies (OR 2.51, 95% CI 2.12-2.98) [10].

The findings of this study aim to provide valuable insights into the frequency of and risk factors for placenta previa among pregnant women. This information could aid hospital administration in better managing such cases and inform public health departments to include these statistics in annual reports, ultimately contributing to global health policies and legislative reforms. Furthermore, early diagnosis and effective management strategies may help prevent the adverse outcomes associated with placenta previa.

## Materials and methods

This cross-sectional study took place in the Gynecology and Obstetrics department at Ayub Teaching Hospital in Abbottabad, Pakistan, from August 2024 to January 2025, and involved 142 pregnant women who were selected using a non-probability consecutive sampling method. The study was approved by the Institutional Medical and Ethics Review Committee of Ayub Teaching Hospital (reference number RC-EA-2024/034). All participants provided informed consent before inclusion in the study and confidentiality of patient data was strictly maintained throughout the research process.

Inclusion and exclusion criteria

We included all pregnant women of gestational age greater than 28 weeks, who visited the Gynecology and Obstetrics department during the study period. Women diagnosed with placenta previa via ultrasound were included. However, those with multiple pregnancies, known uterine anomalies, or diagnosed with placental abruption were excluded to keep the results clear of confounding factors.

Data collection

To gather relevant demographic and clinical information, we created a structured proforma. This collected details on age, education, occupation, area of residence, socioeconomic status, and risk factors for placenta previa. We also recorded obstetric history, including gravidity, parity, previous cesarean sections, abortions, and any past instances of placenta previa. Data was collected through direct patient interviews and cross-checked with clinical records to ensure accuracy and completeness. Ultrasound confirmation of placenta previa was standardized and performed by a consultant radiologist to ensure diagnostic accuracy. These findings were further validated against patients' medical records to maintain consistency and reliability of diagnosis.

Data analysis

We analyzed the data using IBM SPSS Statistics, version 22.0 (IBM Corp., Armonk, NY). Categorical variables, such as past cesarean sections, abortions, and age groups, were described in terms of frequencies and percentages. We used the chi-square test to determine associations between these categorical variables and the occurrence of placenta previa, considering a p-value of ≤0.05 as statistically significant. In cases where expected cell counts were less than five, such as prior history of placenta previa, Fisher’s exact test was applied for more accurate analysis. For quantitative variables like age, parity, gravidity, and the number of cesarean sections, we provided descriptions using mean, median, mode, and standard deviation.

## Results

Demographics and frequency of placenta previa

A total of 142 pregnant women were included in the study, with a mean age of 26.33 ± 5.37 years. The majority (78%, n = 111) were below 35 years of age, while 22% (n = 31) were aged 35 years and above. The frequency of placenta previa was 6.3% (n = 9). Among these cases, 77.8% (n = 7) were below 35 years of age, and 22.2% (n = 2) were aged 35 years and above.

Obstetric history and risk associations

The occurrence of placenta previa was higher among women with multigravidity (three or more pregnancies) and multiparity (three or more deliveries). A total of 77.8% (n = 7) of placenta previa cases were multigravid compared to 31.6% (n = 42) in those without placenta previa (p = 0.01). Similarly, 66.7% (n = 6) of the placenta previa group had multiparity, versus 27.1% (n = 36) in the non-previa group (p = 0.026).

A significant association was also found with previous cesarean sections: 55.6% (n = 5) of placenta previa cases had a history of cesarean section, compared to 21.1% (n = 28) of those without (p = 0.015). A past history of placenta previa notably increased the risk, with 66.6% (n = 6) reporting previous episodes, compared to 2.3% (n = 3) in the control group (p = 0.006).

Multivariate logistic regression analysis

**Figure 1:**
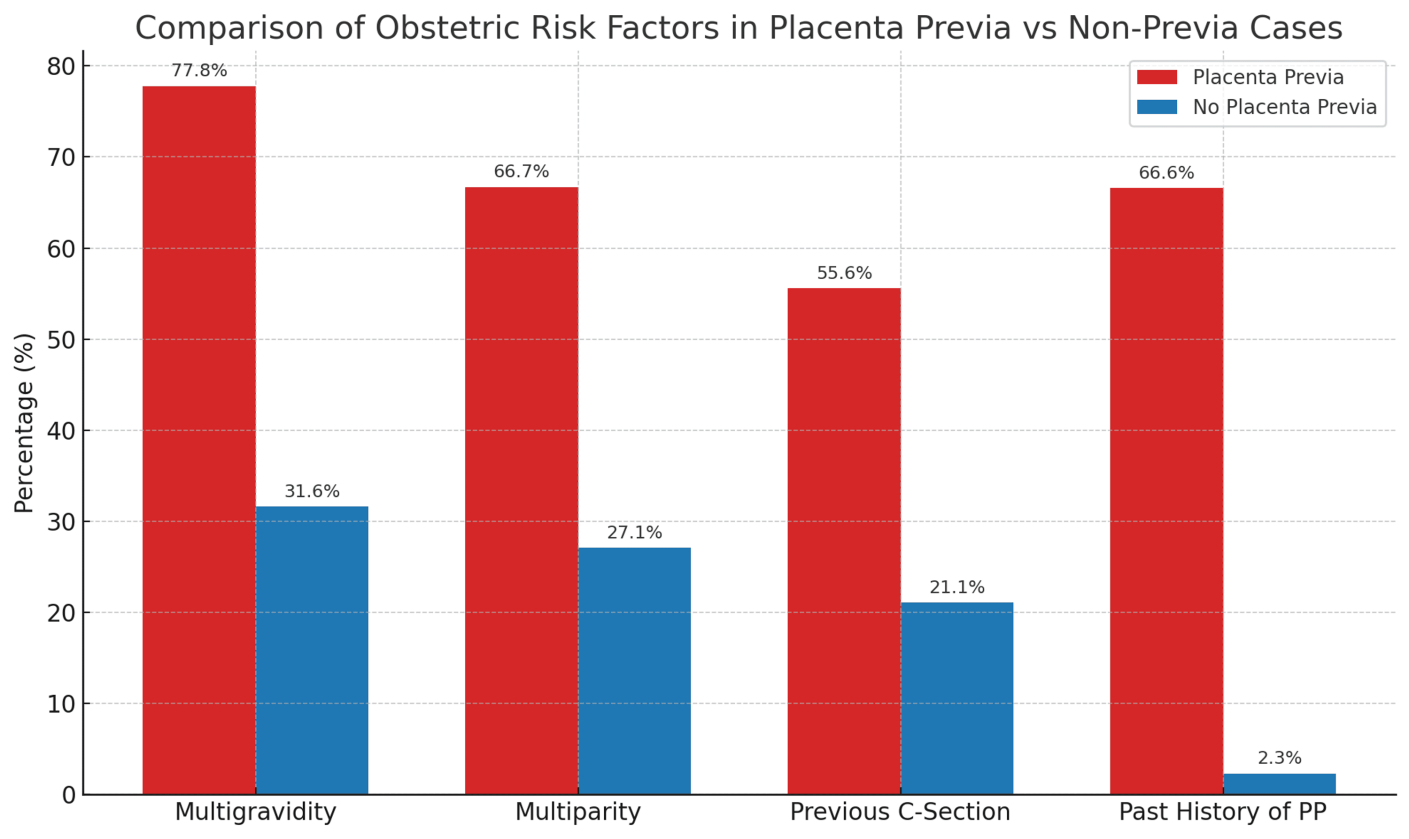
Association of multigravidity, multiparity, prior cesarean sections, and previous placenta previa (PP) with the risk of developing placenta previa

A binary logistic regression model was applied to adjust for potential confounders. After adjusting for maternal age, multigravidity, and previous cesarean section, past history of placenta previa remained the strongest independent predictor, with an odds ratio (OR) of 28.5 (95% CI 5.4-151.3, p < 0.001). Multigravidity was also significantly associated with placenta previa (OR = 4.6, 95% CI 1.1-18.7, p = 0.03), while previous cesarean section retained a borderline significance (OR = 3.9, 95% CI 0.9-16.4, p = 0.07). Figure 1 shows the distribution of key obstetric risk factors, such as multigravidity, multiparity, prior cesarean section, and past history of placenta previa, among women with and without placenta previa. It highlights higher frequencies of multigravidity, multiparity, previous cesarean sections, and a past history of placenta previa in the affected group. These differences are visually evident and align with the statistical associations observed in both the chi-square and logistic regression analyses, emphasizing the clinical relevance of these factors in predicting placenta previa.

Clinical presentation and management

All patients with placenta previa (100%, n = 9) presented with painless vaginal bleeding during the second or third trimester. Of these, 77.8% (n = 7) experienced recurrent bleeding, while 22.2% (n = 2) had a single major bleeding episode; 66.7% (n = 6) required inpatient care for stabilization, while 33.3% (n = 3) were managed on an outpatient basis with close follow-up.

Delivery method and neonatal outcomes

The majority of placenta previa cases (88.9%, n = 8) were delivered via cesarean section, while vaginal delivery occurred in only one marginal placenta previa patient (11.1%, n = 1) under strict monitoring. Blood transfusion was needed in 44.4% (n = 4) of placenta previa patients compared to 8.3% (n = 11) in non-previa patients (p = 0.004).

Regarding neonatal outcomes, 33.3% (n = 3) resulted in preterm births, with 22.2% (n = 2) requiring NICU admission. Most neonates had normal Apgar scores, although one neonate required resuscitation. No cases of intrauterine fetal demise were observed. Table 1 summarizes the distribution of key demographic and obstetric risk factors in relation to placenta previa.

**Table 1:** Association of risk factors and clinical outcomes with placenta previa

Characteristics	Placenta previa (n = 9)	No placenta previa (n = 133)	p-value
Mean age (years)	26.33 ± 5.37	27.01 ± 5.12	0.521
Age group (<35 years)	7 (77.8%)	104 (78.2%)	0.973
Age group (≥35 years)	2 (22.2%)	29 (21.8%)	0.973
Multigravidity (≥3 pregnancies)	7 (77.8%)	42 (31.6%)	0.010
Multiparity (≥3 deliveries)	6 (66.7%)	36 (27.1%)	0.026
Previous C-section	5 (55.6%)	28 (21.1%)	0.015
History of placenta previa	6 (66.6%)	3 (2.3%)	0.006
Painless vaginal bleeding	9 (100%)	0 (0%)	—
Cesarean section delivery	8 (88.9%)	31 (23.3%)	<0.001
Vaginal delivery	1 (11.1%)	102 (76.7%)	<0.001
Blood transfusion requirement	4 (44.4%)	11 (8.3%)	0.004
Preterm births	3 (33.3%)	9 (6.8%)	0.021
NICU admission	2 (22.2%)	7 (5.3%)	0.034
Intrauterine fetal demise (IUFD)	0 (0%)	3 (2.3%)	0.603

In this study, the frequency of PP was observed to be 6.3% among the participants. Significant associations were identified between placenta previa and several risk factors, including multigravidity, multiparity, previous cesarean sections, and a history of placenta previa. The majority of cases (88.9%) resulted in cesarean deliveries due to the heightened risk of bleeding and maternal complications, with only 11.1% achieving vaginal delivery under strict monitoring. Adverse fetal outcomes were also noted, with 33.3% of the placenta previa cases resulting in preterm births and 22.2% requiring NICU admission. Despite these risks, no cases of intrauterine fetal demise (IUFD) were reported in the PP group.

## Discussion

The prevalence of placenta previa among pregnant women attending the gynecology unit was found to be 6.3%, which is considerably higher compared to the global prevalence of 0.5% and the Asian prevalence of 1.2% [3]. In comparison to other studies, its prevalence was reported as 0.6% in a retrospective study conducted in CMH Kharian, Pakistan, from 2015 to 2018 [5], and 1.8% in a cohort study from Gulbarga, India [6]. The higher prevalence in our study may be attributed to factors such as the study being conducted in a tertiary care setting, small sample size, short study duration, and non-probability sampling.

The significant risk factors identified in our study were multiparity, a history of miscarriages, and a history of previously diagnosed placenta previa. These findings align with a study from Northern Tanzania, which highlighted advanced maternal age, gynecological disease, alcohol use during pregnancy, previous cesarean sections, history of uterine scars, and fetal malpresentation as significant risk factors [8]. A meta-analysis of observational studies also identified advancing maternal age, multiparity, previous cesarean sections, abortions, smoking, alcohol, and cocaine use as risk factors for placenta previa [11].

In our study, 22% of women with placenta previa were aged 35 years and above, while 78% were below 35 years old (OR = 2.42, 95% CI 0.45-12.85), which was statistically insignificant. This differs from a meta-analysis (covering 2005-2015) that found a strong association between maternal age of 35 years and above and PP (OR = 3.16, 95% CI 2.79-3.57) [12]. Similarly, a study from Nigeria reported that 77.3% of placenta previa cases were found in women aged 35 and above [13]. The higher association of PP with younger women in our study could be due to early marriages, higher parity at a younger age, and study limitations such as sample size and duration.

Our study found that 44.4% of placenta previa cases were associated with multigravidity (G > 1 and G ≤ 4), while 55.6% were linked with grand multigravidity (p = 0.01), and 37% were linked with multiparity (p = 0.028), indicating significant associations. In comparison, a study conducted at CMH Lahore reported that 81.1% of placenta previa cases were associated with multigravidity and multiparity [14].

In our study, 67% of women with placenta previa had a positive history of previous cesarean sections, making them three to four times more at risk. This aligns with findings from a cross-sectional descriptive study from CMH Lahore, where 62.59% of placenta previa cases had a history of cesarean sections [14]. Globally, the risk of placenta previa increases to 10% from 0.26% with a prior cesarean section [15].

A history of induced abortion was present in 11.1% of women with placenta previa in our study (OR = 2.646, 95% CI 0.283-24.712). This is consistent with findings from a study in an obstetric care hospital in Western Washington, where induced abortion by sharp curettage was linked to an increased risk of placenta previa (OR = 2.9, 95% CI 1.0-8.5, for ≥3 abortions) [16].

A retrospective cohort study in the UKM Medical Centre in Kuala Lumpur, Malaysia, found that primary gravidity with a history of uterine disease, such as endometriosis, had a higher risk of placenta previa (21% vs. 6% in non-primary gravidity) [17]. However, in our study, no women with placenta previa had uterine diseases like endometriosis.

In our findings, 22% of women with placenta previa had a past history of the condition, making them nine times more likely to have a recurrence compared to the general population. This is higher than the recurrence rate of 3.2% (six times more risk) reported in a study from Beilinson Medical Center, Israel [18].

While previous uterine surgery, assisted reproductive techniques, multiple gestations, and smoking are documented as risk factors for placenta previa in various studies, our study did not find any significant association with these factors.

This study has some limitations that should be considered. Firstly, the sample size of 142 participants may not be large enough to accurately reflect the wider population, which could impact the generalizability of the findings. Additionally, since the research was conducted at a single location, the results may not be fully applicable to other regions or healthcare settings. Moreover, the cross-sectional design of the study limits the ability to establish cause-and-effect relationships between placenta previa and its associated risk factors. The use of non-probability consecutive sampling, while suitable for this setting, also limits the generalizability of the results and should be acknowledged as a methodological limitation. Finally, relying on patients’ self-reported histories for data collection may introduce recall bias. To strengthen these findings, future research should involve larger, multi-center populations and utilize prospective study designs.

## Conclusions

In this study, we found that the occurrence of placenta previa was notably higher than what is typically seen worldwide. Some key risk factors that stood out included having a history of placenta previa, being a grand multipara (which means having given birth five times or more), and experiencing multiple pregnancies. We also noted other possible risk factors, such a history of cesarean sections and the age of the mother. Interestingly, most cases of placenta previa were observed in women under 35, and a significant number of these women had at least one previous cesarean delivery. These results indicate that a mix of having multiple pregnancies, being younger, and surgical history could elevate the risk of placenta previa among expectant mothers.
